# Therapeutic effect of sustained release of aloe emodin via novel grafted polymer in electrospun nanofiber membranes on peritendinous adhesions

**DOI:** 10.3389/fbioe.2026.1780630

**Published:** 2026-04-21

**Authors:** Xinpeng Dong, Yuan Wang, Yi Li, Xin Wang, Qing Yu, Changhao Han, Jiayi Wang, Shen Liu, Jinglei Wu, Shenghe Liu

**Affiliations:** 1 Department of Orthopaedics, Shanghai Sixth People’s Hospital Affiliated to Shanghai Jiao Tong University School of Medicine, Shanghai, China; 2 Shanghai Engineering Research Center of Nano-Biomaterials and Regenerative Medicine, College of Biological Science and Medical Engineering, Donghua University, Shanghai, China; 3 Department of Hand Microsurgery and Plastic Reconstructive Surgery, Ningbo No. 6 Hospital, Ningbo, China

**Keywords:** aloe emodin, electrospun nanofiber membranes(ENM), fibrosis, peritendinous adhesions, TGFβ1/COL3

## Abstract

**Introduction:**

Tendon injuries are prevalent musculoskeletal conditions in clinical settings, affecting various regions such as the rotator cuff tendons, forearm flexor/extensor tendons, finger flexor tendons, and Achilles tendon. While surgery is an effective treatment, it often fails to balance intrinsic and extrinsic healing processes, leading to abnormal cell activation, proliferation, and migration, resulting in collagen deposition at the injury site. This pathological fibrosis causes severe peritendinous adhesions, posing a therapeutic challenge. Traditional nonsteroidal anti-inflammatory drugs (NSAIDs) and implantable membranes have limited long-term efficacy and come with various side effects. Literature indicates that Aloe emodin(AE) can potentially inhibit multiple known pro-inflammatory pathways, including NF-κB, MAPK, p38, and ERK, directly or indirectly reducing oxidative stress. In the field of fibrosis, Aloe emodin regulates the TGFβ/Smad signaling pathway, downregulates extracellular matrix gene transcription, inhibits collagen deposition, improves cardiac function and myocardial fibrosis. Based on these findings, we hypothesize that Aloe emodin may serve as an effective therapeutic agent for improving peritendinous adhesions.

**Methods:**

This study involved grafting Aloe emodin onto polylactic acid via esterification to create a polylactic acid ‐ Aloe emodin conjugate compound. Using electrospinning technology, a novel polylactic acid-Aloe emodin conjugate electrospun nanofiber membrane (PCA) was developed and characterized, and its role in preventing peritendinous adhesions was thoroughly validated. We validated and systematically evaluated the anti-adhesive capability of the new electrospun membrane through in vitro and in vivo experiments in rats.

**Results:**

Experimental results demonstrate that the electrospun membrane of polylactic acid-Aloe emodin grafted material exhibits excellent mechanical properties and hydrophilicity, and can achieve localized targeted release of damaged tissues after treatment with lipase. Compared with polylactic acid-Aloe emodin hybrid electrospun membrane (PBA), PCA exhibits superior anti-adhesion properties and maintains longer-lasting therapeutic effects. In vitro studies showed that PCA effectively reduced fibroblast activity, inhibiting their proliferation, adhesion, and PA formation. In vivo experiments confirmed that PCA could effectively wrap around surgically treated tendons and inhibit the TGFβ1/COLIII signaling pathway, significantly reducing peritendinous adhesions in rats, offering a new approach for PA treatment.

**Discussion:**

In this study, we designed and manufactured a novel polylactic acid - Aloe emodin conjugate electrospun nanofiber membrane. We validated and systematically evaluated the anti-adhesive capability of the new electrospun membrane through in vitro and in vivo experiments in rats. In summary, our research proposed a new approach for the treatment of tendon adhesions.

## 1 Introduction

Tendon injuries are a common orthopedic condition seen in clinical practice, especially in emergency settings. Surgical intervention, although an effective treatment for tendon injuries (Khan and Carey Smith, 2010), often fails to restore the balance between intrinsic and extrinsic healing, leading to abnormal proliferation, migration, and collagen deposition of tendon-related cells (Linderman et al., 2016; Graham et al., 2019). This pathological fibrosis results in tendon adhesion, which can cause patients to experience pain, swelling, and limited range of motion. In severe cases, it can ultimately lead to functional loss (de Putter et al., 2012). Severe peritendinous adhesion(PA) can significantly diminish patients’ quality of life and increase societal medical burdens, highlighting the need for innovative and effective treatment interventions (Maffulli et al., 2003).

**SCHEME 1:**
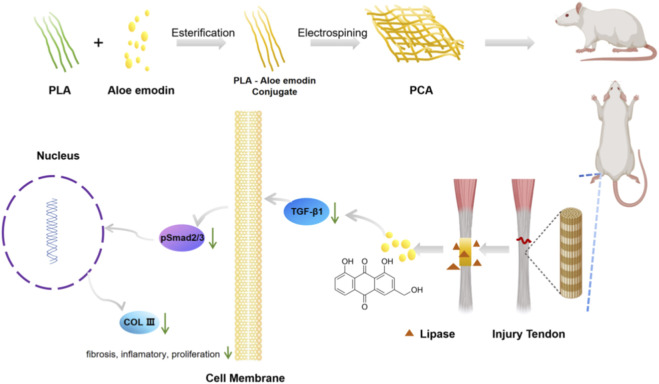
PLA - Aloe emodin Conjugate were synthesized by grafting the hydroxyl of Aloe emodin to the carboxyl of PLA through esterification, and then electrospinning them into nanofiber membranes. Aloe emodin released in respond to lipase to attenuate PA formation by inhibite TGFβ 1/COLIII signaling pathway.

Regarding drug treatment, literature from the 1980s reported the use of nonsteroidal anti-inflammatory drugs (NSAIDs), such as ibuprofen, injected into injured areas to reduce the expression of pro-inflammatory factors like arachidonic acid, thereby minimizing local Post-trauma damage. However, the extensive and unconventional use of NSAIDs has significantly increased the incidence of digestive, cardiovascular, and urinary system diseases among patients (Moore et al., 2019). Given that peritendinous adhesions are closely linked to inflammation, oxidative stress, and excessive fibrosis, recent research has explored clinical treatments through multiple pathways, including anti-inflammatory, antioxidant, antifibrotic, and gene therapy. Nonetheless, clinical application has been challenging due to issues such as numerous side effects, lack of sustained therapeutic effects, and recurrence of adhesion (Wang et al., 2023; Cordiano et al., 2025; Harlev et al., 2012). Therefore, identifying a novel, low-side-effect small-molecule anti-adhesion drug is crucial for treating peritendinous adhesions. Traditionally, Aloe vera is widely used for its skin protection, wound healing, and ability to regulate blood sugar and cholesterol levels (Sánchez et al., 2020). One of its effective bioactive components, Aloe emodin, exhibits significant anti-inflammatory, antioxidant, and immune-regulating properties. The anti-inflammatory effects of Aloe emodin are attributed to its potent inhibition of multiple known pro-inflammatory pathways, such as NF-kB, MAPK, p38, ERK, and JAK. Additionally, Aloe emodin can reduce oxidative stress through direct clearance and indirect pathways (stimulating Nrf2 nuclear translocation and upregulating the expression of various antioxidant and cell-protective genes) (Brebels and Mignon, 2022; Ai et al., 2024; Ren et al., 2024; Xie et al., 2023; Xu et al., 2023; Song et al., 2024; Xie et al., 2025; Dai et al., 2025). Reports indicate its ability to improve fibrosis in multiple organs, including the heart, liver, and kidney (Dou et al., 2019; Zhou et al., 2021; Kam et al., 2024). Literature suggests that Aloe emodin can regulate the TGFβ/Smad signaling pathway, downregulate the transcription of extracellular matrix genes, inhibit collagen deposition, and improve cardiac function and myocardial fibrosis. Consequently, we speculate that Aloe emodin could be an effective drug for improving peritendinous adhesion (Dou et al., 2019; Yu et al., 2023).

To replicate the extracellular matrix components under natural physiological conditions, researchers often employ various scaffold materials for tissue repair and regeneration (Kam et al., 2024; Tan et al., 2025; Cai et al., 2025) Among these options, electrospun nanofibers have gained significant attention due to their versatile functionalities, adjustable fiber morphology, size, and orientation, precise therapeutic loading, and potential for large-scale, simple, and cost-effective manufacturing (Kam et al., 2024; Qu et al., 2025; Cho et al., 2025; Guo et al., 2025). These nanofibers have been successfully used in regenerative therapies for multiple organs and tissues, such as nerves, blood vessels, bones, skin, liver, and kidneys (Kam et al., 2024; Yang A. et al., 2025; Liu et al., 2025; Ji et al., 2025; Yang et al., 2025b; Lee et al., 2024a; Guo et al., 2024; Shanto et al., 2024; Sun et al., 2024; Lee et al., 2024b; Carrabba et al., 2023). However, the rapid release and insufficient sustained release of the drugs and therapeutic agents they carry necessitate the development of new electrospun nanofiber membranes to better meet therapeutic needs (Su et al., 2021; He et al., 2025).

In this study, we designed and manufactured a novel polylactic acid - Aloe emodin conjugate electrospun nanofiber membrane. Aloe emodin was conjugated to polylactic acid through esterification reactions to prepare the polylactic acid - Aloe emodin conjugate compound. Subsequently, the compound was fabricated into a nanofiber membrane using electrospinning technology. We validated and systematically evaluated the anti-adhesive capability of the new electrospun membrane through *in vitro* and *in vivo* experiments in rats. In summary, our research proposed a new approach for the treatment of tendon adhesions (Scheme 1).

## 2 Result

### 2.1 Characterization of different drug-loaded ENMs

**FIGURE 1:**
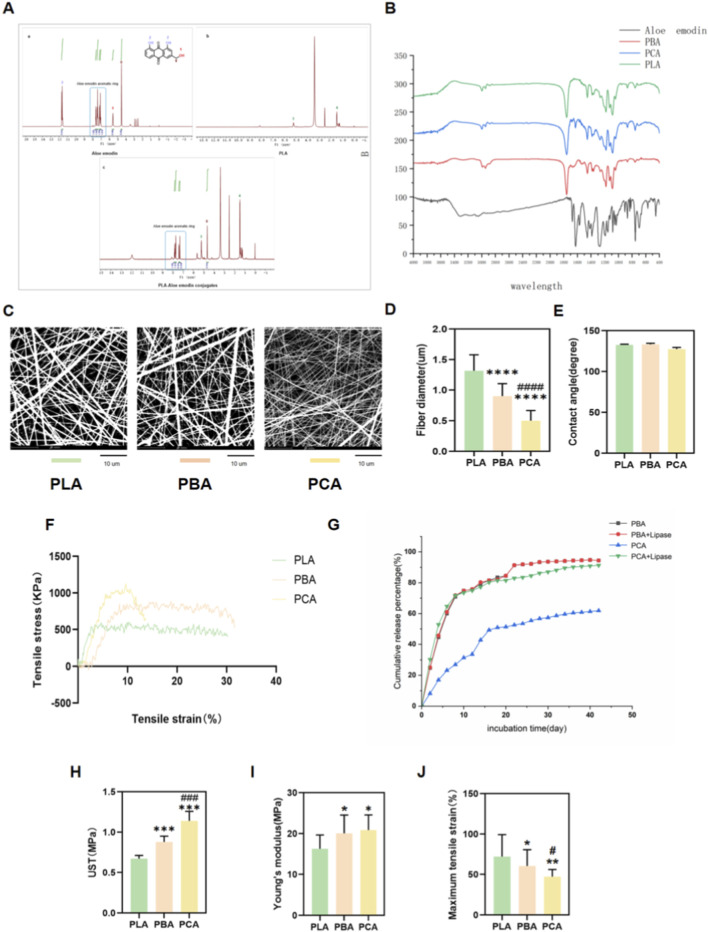
Characterization of different drug loaded ENMs. **(A)** H-Nuclear Magnetic Resonance Spectroscopy of polymer. **(B)** FTIR of all three electrospun nanofiber membranes. **(C)** Scanning electron microscope (SEM) macrographs and **(D)** fiber diameters of PLA, PCA, and PBA. Images quantifying the statistical distribution of **(E)** water contact angles, **(F)** mechanical analysis of stress/stain for PLA, PCA, and PBA, **(G)** Cumulative release percentage of Aloe emodin *in vitro* after incubation in PBS at 37 °C. The integral value in the drug release curve = (sum of drug release amounts on all days before this time point)/(total drug release over 42 days) * 100% **(H)** UST analysis. **(I)** Young’s modulus and **(J)** maximum tensile strain (value by mean ± SD, *p < 0.05, **p < 0.01, ***p < 0.001, ****p < 0.0001. The symbols “*” and “#” indicate intergroup comparisons with the PLA group and PBA group, respectively. (n = 6).

In this study, PLA - Aloe emodin conjugates were prepared by electrospinning and characterized. Through esterification, we successfully synthesized a novel PLA-Aloe emodin graft material. To demonstrate the successful grafting of alloplastin, we subsequently analyzed the specific composition of the material. Fourier transform infrared spectroscopy (FTIR) revealed strong peaks at 1767 and 1,546 cm^−1^ corresponding to the tC = C bonds of anthraquinone carbonyl groups and aromatic groups, respectively (Figure 1B). Structural analysis of Aloe emodin showed three distinct characteristic peaks in addition to the hydrogen atoms in the aromatic ring, which were labeled as 0, 1, and 2 (Figure 1A). Similarly, PLA exhibited two distinct characteristic peaks labeled as 3 and 4 (Figures 1A,B). In the PLA-Aloe emodin graft material, characteristic peaks of both PLA and Aloe emodin were observed simultaneously, with the hydroxyl groups almost completely absent, indicating successful esterification(Figures 1A,C). Based on the integration values of the characteristic proton peaks—specifically, the aromatic proton peak of Aloe emodin and the methyl peak (-CH_3_) of PLA—with areas of 1.00 and 2.53, respectively, the calculated degree of substitution is 60%(Supplementary Figure S4).

Statistical results showed that PLA, PBA, and PCA electrospun nanofibers generally exhibited a round, porous, and randomly arranged structure, although their electrospun fiber diameters were significantly different (Figure 1C). PCA nanofibers had the smallest diameter, resulting in the smallest fiber-to-fiber distance (Figure 1D). The water contact angle results of PLA, PBA, and PCA showed no significant differences(Supplementary Figure S6). The results showed that grafting Aloe emodin into the molecular chain of polylactic acid did not affect the original hydrophilic properties of the polylactic acid electrospun membrane, which was an effective solution to the low water solubility of Aloe emodin(Figure 1E). We also evaluated their mechanical properties by testing elastic modulus, tensile strength, and strain (Figures 1F,H,I). PBA and PCA exhibited similar Young’s modulus (20.098 and 20.8856 MPa), both higher than the PLA group (16.320 MPa). In the ultimate tensile strength (UTS), PCA showed relatively superior tensile strength (1.140 MPa) compared to PLA and PBA (0.674 and 0.881 MPa, respectively). The fracture elongation indicated that the PCA group was more brittle, with a smaller deformation range after stretching. The release curve showed that Aloe emodin had a burst release in the first 10 days in PBA and PCA, followed by a sustained release of about 14 days, using lipase. Aloe emodin in PBD was exhausted around day 30, while PCA could continuously release Aloe emodin until day 42 (Figure 1G).

### 2.2 PCA inhibits fibroblast proliferation and adhesion in vitro

#### 2.2.1 PCA inhibits fibroblast proliferation

**FIGURE 2:**
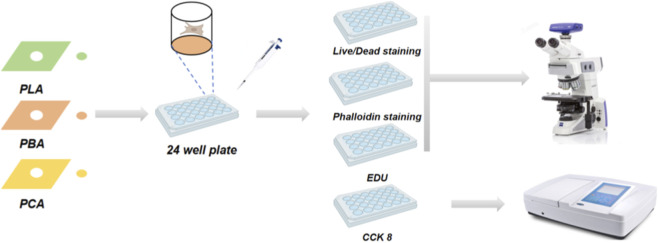
Experimental process for assessment of fibroblast proliferation and adhesion ability *in vitro*.

**FIGURE 3:**
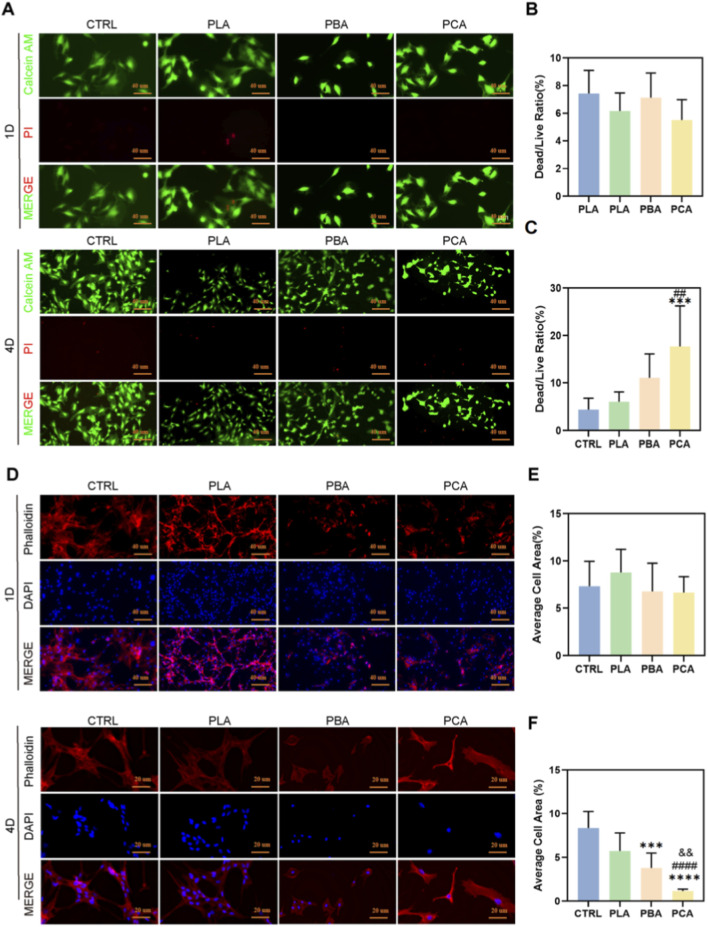
Biological Assessment *in Vitro*. **(A)** Live/dead fluorescence staining of 208F after being cultured for 1 and 4 d with lipase. **(B,C)** Quantitative results of the dead/live cell ratios in each group on days 1 and 4. **(D)** Representative images of phalloidin staining in each group on days 1 and 4 with lipase. **(E,F)** Quantitative results of the average cell area from phalloidin staining in each group on days 1 and 4. Value by mean ± SD, *p < 0.05, **p < 0.01, ***p < 0.001, ****p < 0.0001. The symbols “*”, “#” and “&” indicate intergroup comparisons with the control group (CTRL), PLA group, and PBA group, respectively. (n = 6).

This study first assessed the effects of different materials on fibroblast 208F cells using live/dead staining (Figure 2) at two time points (day 1 and day 4) (Figure 3A). On day 1, there were no significant differences in the live/dead cell ratios among the four groups—Control (CTRL), PLA alone (PLA), PLA blended with Aloe emodin (PBA), and PLA grafted with Aloe emodin (PCA), indicating comparable initial cell seeding densities across all groups (Figure 3B). By day 4, however, the dead/live ratios in the PBA and PCA groups were significantly higher than those in the CTRL and PLA groups, suggesting that Aloe emodin exerts cytotoxic effects on fibroblasts (Figure 3C).

**FIGURE 4:**
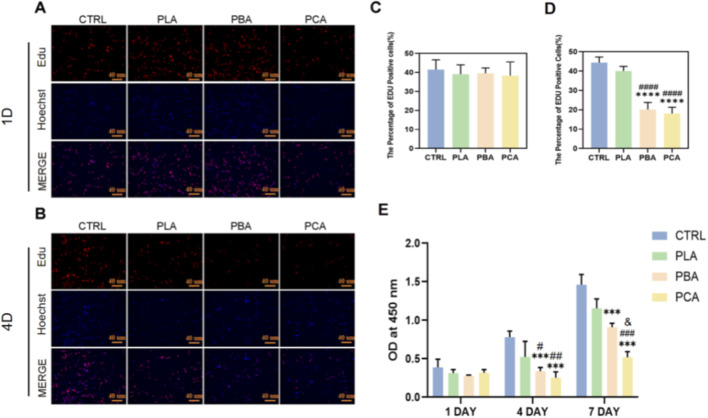
Biological Assessment *in Vitro*. **(A,B)** EDU staining of 208F after being cultured for 1 and 4 d with lipase. **(C,D)** Quantitative results of the percentage of EDU Positive cell ratios in each group on days 1 and 4. **(E)** Proliferation ability of fibroblasts cultured on different membranes. Value by mean ± SD, *p < 0.05, **p < 0.01, ***p < 0.001, ****p < 0.0001. The symbols “*”, “#” and “&” indicate intergroup comparisons with the control group (CTRL), PLA group, and PBA group, respectively. (n = 6).

To detect the proliferative activity of fibroblasts between different treatment groups during cultivation, we used the EDU staining kit to stain the cells in culture for proliferation. The results showed that on day 4, the ratio of proliferating fibroblasts in the PBA and PCA groups was significantly lower than that in the control group and PLA-treated group, while there was no significant difference between the PBA and PCA groups. This indicates that Aloe emodin has a clear inhibitory effect on fibroblast proliferation (Figures 4A–D).

#### 2.2.2 PCA inhibits fibroblast adhesion

To further investigate cell morphology, phalloidin staining was performed across all groups (Figure 3D). Quantitative analysis demonstrated that average cell areas were similar across groups on day 1, again confirming comparable initial seeding densities (Figure 3E). By day 4, the average cell area in the PCA group was markedly smaller than in the other three groups, indicating that aloe emodin significantly inhibits fibroblast spreading and growth. Notably, PLA grafted with aloe emodin (PCA) exhibited a stronger inhibitory effect on 208F cells compared with PLA directly blended with aloe emodin (PBA) (Figure 3F).

In addition, cell viability was evaluated using the CCK-8 assay, with OD values measured on days 1, 4, and 7 (Figure 4E). On day 1, OD values were comparable across all groups, consistent with the initial seeding results. By days 4 and 7, OD values in the PCA group were significantly lower than in the other three groups, in agreement with the live/dead and phalloidin staining findings. These results further confirm that Aloe emodin strongly suppresses fibroblast proliferation, with grafted PLA (PCA) exerting a more pronounced inhibitory effect than directly blended PLA (PBA).

### 2.3 PCA inhibits PA formation in vivo

#### 2.3.1 PCA electrospun membrane prevents PAF

**FIGURE 5:**
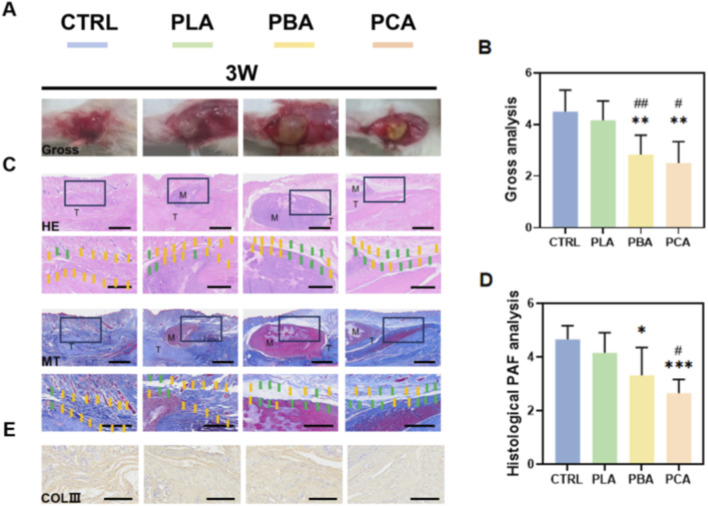
*In vivo* analysis of PCA membranes in tendon injury (TI) rat models. **(A)** Gross observation of PAF on post-operative week 3. **(B)** Gross analysis of PAF on post-operative week 3. **(C)** Hematoxylin-eosin (HE) and masson trichrome (MT) staining of TI tissues on week 3. Black boxes indicate the magnified view. Scale bar is 2 mm for non-magnified images and 1 mm for magnified images. **(D)** Histological scoring for PAF on week 3. **(E)** Immunohistochemical (IHC) staining of Collagen III (ColIII) on week 3. Results are presented as means ± SD. Statistical significance was calculated by one-way ANOVA. *p < 0.05, **p < 0.01, ***p < 0.001. The symbols “*”, “#” and “&” indicate intergroup comparisons with the control group (CTRL), PLA group, and PBA group, respectively. (n = 6). Scale bar is 200 μm.Yellow arrows indicate adhesions, while green arrows indicate no adhesion tissue was observed.

**FIGURE 6:**
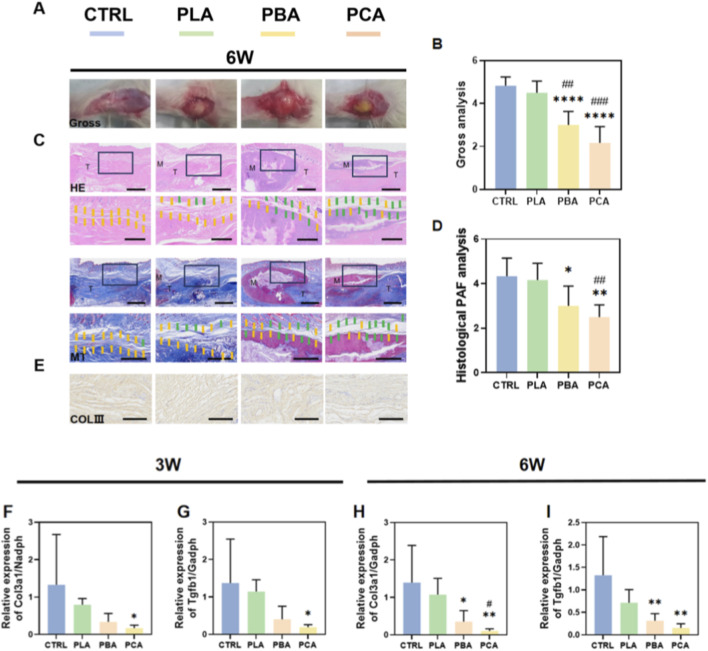
*In vivo* analysis of PCA membranes in tendon injury (TI) rat models. **(A)** Gross observation of PAF on post-operative week 6. **(B)** Gross analysis of PAF on post-operative week 6. **(C)** Hematoxylin-eosin (HE) and masson trichrome (MT) staining of TI tissues on week 6. Black boxes indicate the magnified view. Scale bar is 2 mm for non-magnified images and 1 mm for magnified images. **(D)** Histological scoring for PAF on week 6. **(E)** Immunohistochemical (IHC) staining of Collagen III (ColIII) on week 6. **(F,G)** qPCR analysis of ColIII, Tgfb1 of PAF tissue on week 3. **(H,I)** qPCR analysis of Col3a1, Tgfb1 of PAF tissue on week 6. Results are presented as means ± SD. Statistical significance was calculated by one-way ANOVA. *p < 0.05, **p < 0.01, ***p < 0.001, ****p < 0.0001. The symbols “*”, “#” and “&” indicate intergroup comparisons with the control group (CTRL), PLA group, and PBA group, respectively. (n = 6). Scale bar is 200 μm. Yellow arrows indicate adhesions, while green arrows indicate no adhesion tissue was observed.

To investigate the *in vivo* capacity of PCA membranes on the prevention of PAF, we further applied the membrane to a tendon injury (TI) rat model. Blank, PBA, and PLA electrospun membranes were used to compare effects. PAF was evaluated on week 3 and week 6 (3W and 6W) postoperatively. Gross observation revealed no infection or ulcers at the implantation site. Gross analysis of adhesion showed that the Control and treated tendons were highly adherent to the surrounding tissue, as the detacher could not easily penetrate through the PAF tissue. In contrast, the detacher could easily penetrate the PAF tissue within the PBA and PCA treated tendons on 3W and 6W (Figures 5A, 6A).

The histological analysis of healed TI tissue was further analyzed via HE and Masson staining. In the Control and treated groups, there was a dense and stiff amount of adhesion tissue formed around the injured tendon by 3W. This indicated the reason for the increased gross adhesion scoring rate within these groups. Applying PBA membrane treatment reduced the amount of PAF formation compared to Control and PLA treatment. As for PCA treatment, the adhesion formation was significantly inhibited between the wounded tendon and adjacent tissue in contrast to the other treated groups (Figures 5B–D). By 6W, the anti-PAF effects treated by PCA membranes were further enhanced compared to 3W, as the other treated groups had no significant difference against their results on 3W (Figures 6B,C). The histological scoring further verified the observations (Figures 5D, 6D). In addition, to evaluate the effect of PCA implantation on normal tendon healing after Achilles tendon injury in rats, we used a universal tensile testing machine to assess the tensile strength of Achilles tendon samples at different time points. The experimental results indicated that the implantation of different materials of electrospun membranes did not significantly alter the mechanical strength of Achilles tendon healing, suggesting its limited role in the healing process of deep Achilles tendon tissues(Supplementary Figure S9).

#### 2.3.2 PCA inhibits the TGFβ1 - pSmad2/3 - COLIII pathway to reduce collagen deposition

As PCA membranes had a significant effect on the inhibition of PAF, immunohistochemical (IHC) staining of Collagen III (COLIII), the major type of extracellular matrix (ECM) that accumulates after injury, was used to evaluate its effect on the inhibition of collagen deposition. COLIII is also the main ECM that composes the forming of adhesion tissue. IHC scoring of Col III indicated that PCA and PBA membrane-treated tendons were significantly reduced compared to blank control and PLA treatment (Figures 5E). We further investigated the underlying mechanism of the decreased COLIII deposition. As Aloe emodin has a major effect on the TGFβ pathway, the key signaling molecules (TGFβ1 and COLIII) within the PAF tissue were analyzed. Results showed that treated tendons had significantly downregulated expression of TGFβ1 and COLIII in the PAF tissue on 3W and 6W. (Figures 6F–I). These results indicated that PCA membrane could significantly reduce PAF.

**FIGURE 7:**
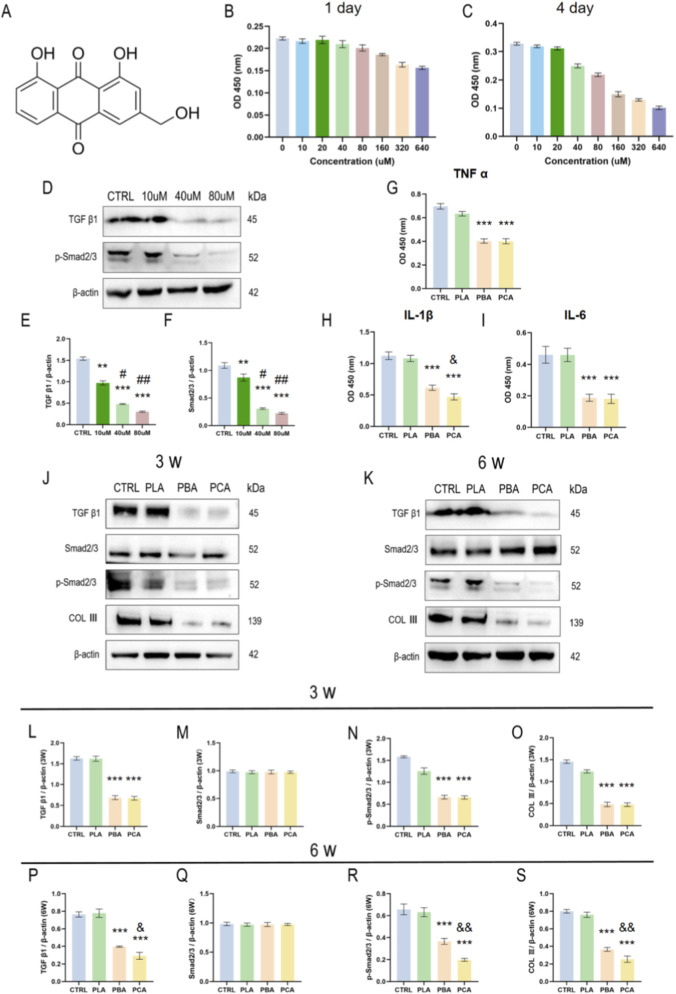
Mechanism of TGFβ1 pathway in tendon injury (TI) rat models of PAF treated with PCA membranes. **(A)** Chemical molecular structure of Aloe emodin. **(B)** Fibroblast viability detected using the CCK-8 kit after 1-day treatment with varying concentrations of aloe emollient medium; **(C)** Fibroblast viability detected using the CCK-8 kit after 4-day treatment with varying concentrations of aloe emollient medium; **(D–F)** Western blot analysis of TGFβ1 and pSmad2/3 protein expression in fibroblasts after incubation with 0, 10,40, and 80 μM aloe emollient; **(G–I)** Levels of inflammatory cytokines (TNFα, IL-1β, IL-6) in macrophage cells after 4-day co-incubation with different nanofiber membranes and LPS. **(J)** Expression levels of TGFβ1, Smad2/3, pSmad2/3, and COLIII protein in peritendinous adhesion tissues of rat Achilles tendon injury intervention model at 3 weeks; (n = 3) **(K)** Expression levels of TGFβ1, Smad2/3, pSmad2/3, and COLIII protein in peritendinous adhesion tissues of rat Achilles tendon injury intervention model at 6 weeks; (n = 3) **(L–O)** Statistical analysis of expression levels of TGFβ1, Smad2/3, pSmad2/3, and COLIII protein in peritendinous adhesion tissues of rat Achilles tendon injury intervention model at 3 weeks; **(P–S)** Statistical analysis of expression levels of TGFβ1, Smad2/3, pSmad2/3, and COLIII protein in peritendinous adhesion tissues of rat Achilles tendon injury intervention model at 6 weeks. Results are presented as means ± SD. Statistical significance was calculated by one-way ANOVA. *p < 0.05, **p < 0.01, ***p < 0.001, ****p < 0.0001. “*”, “#” and “&” indicate intergroup comparisons with the control group (CTRL), PLA group, and PBA group, respectively. (n = 6).

After 1 day treatment with Aloe emodin (Figure 7A) at different concentrations in the culture medium, fibroblast activity was detected using the CCK-8 kit. It was observed that cell viability began to decline gradually when the drug concentration reached or exceeded 40 μM (Figure 7B). After 4 days of treatment, significant differences in OD 450 signals were detected among all treatment groups, with 40 μM remaining the critical concentration for this differential effect (Figure 7C). To further investigate the changes in protein level signaling pathways in fibroblasts after Aloe emodin treatment, we hypothesized that the TGFβ1 axis was the signaling pathway involved in the peritendinous adhesion model, based on previous literature on Aloe emodin therapy. Western blotting was performed using rat fibroblast lines. The experimental results showed that after 4 days of co-culture with 0, 10,40, and 80 μM Aloe emodin, the protein levels of TGFβ1 and pSmad2/3 in fibroblasts were significantly inhibited (Figures 7D–F). Therefore, we ultimately determined 40 μM as the effective and safe critical concentration of Aloe emodin for fibroblast treatment. To explore the effects of Aloe emodin on immune cells associated with peritendinous adhesion, macrophages were selected to detect changes in related inflammatory factors after co-treatment with Aloe emodin and lipopolysaccharide (LPS). The results demonstrated that the secretion of macrophage-associated anti-inflammatory factors, TNFα, IL-1β, and IL-6, was significantly downregulated(Figures 7G–I).

We collected peri-tendinous adhesions from rat Achilles tendon injury models at 3 and 6 weeks, and investigated the specific mechanisms of peri-tendinous adhesions reduction after implantation of different polylactic acid membranes through Western blot experiments. The results demonstrated that at 3 weeks of intervention, significant changes in TGFβ1, pSmad2/3, and COL III protein levels were observed in the Aloe emodin treatment group (PBA and PCA groups), with the classical TGFβ1 pathway being inhibited (Figures 7J,L–O). Further exploration revealed that compared to 3 weeks, the TGFβ1 signaling pathway remained persistently inhibited at 6 weeks in the animal model, indicating its stable and effective antagonistic effects against fibrosis and collagen matrix deposition during tendon healing through sustained slow administration (Figures 7K,P–S). This finding is consistent with the previously reported TGFβ1 and COL III PCR transcription results, confirming that Aloe emodin can inhibit peri-tendinous tissue fibrosis and collagen deposition after tendon injury via the TGFβ1 - pSmad2/3 classical pathway, thereby preventing peri-tendinous adhesion formation. This provides new evidence supporting the hypothesis that Aloe emodin can effectively treat peri-tendinous adhesion disorders.

## 3 Discussion

Tendon-related diseases are common clinical musculoskeletal disorders, covering various parts such as the rotator cuff tendon, forearm flexor/extensor tendons, hand flexor tendons, and Achilles tendon. These diseases are prevalent worldwide, with limited treatment efficacy and increasing social medical burden (Maffulli et al., 2003; Douwes et al., 2025; Wang C. et al., 2025). Due to the natural avascular and aneural nature of tendon tissue, post-traumatic treatment often struggles to balance internal and external healing, leading to a severe cycle of “adhesion-surgery-readhesion”, which can result in tendon re-tears, restricted and stiffened movement at the injury site (Debela et al., 2025; Luo et al., 2025; Timoçin and Özen Atalay, 2026). Given the literature reports on the limitations of existing surgical treatments, side effects of drug treatments, and the recurrence of the disease, our team identified the inadequacies of single-therapy approaches in clinical applications. After thorough consideration, we selected electrospinning technology to prepare electrospun nanofiber membranes as physical barriers for implantation (Tan et al., 2025; Humeres et al., 2024; Miyazawa and Miyazono, 2017; Wang et al., 2014; Yong et al., 2024; Chen et al., 2025; Cai et al., 2025; Ma et al., 2025; Tarin et al., 2025). Prior studies have demonstrated that polymer molecular weight can be modulated through direct and indirect effects (such as influencing conductivity and viscosity) to alter the diameter, morphological characteristics, and mechanical properties of electrospun fibers. Through grafting modification of polymers, the molecular weight of polylactic acid (PLA) grafted materials increases. Characterization of electrospun membranes using spinning mechanisms reveals improved silk-like fiber morphology with reduced diameters. This results in significantly enhanced tensile strength and modulus stiffness of nanofibers while decreasing deformation, which aligns with the experimental observations in our study (Wong et al., 2008; Shin et al., 2006; Tan and Lim, 2004; Wingert et al., 2017; Lim et al., 2008). The results show that the electrospun nanofiber membranes of Polylactic Acid-Aloe emodin Grafted Materials not only improve the mechanical properties of the original Polylactic Acid nanofiber membranes, but also solve the problem of low water solubility and poor drugability of Aloe emodin.

As a traditional therapeutic drug, Aloe emodin has been widely reported for application in systemic diseases such as skin, nerve, vascular, and skeletal disorders due to its antioxidant, anti-fibrotic, and inhibitory effects on cell proliferation and adhesion. In this study, we selected Aloe-emodin as a therapeutic agent, taking into full account its mature purification and manufacturing process, extensive clinical application, A research team from Xiangya School of Medicine at Central South University discovered that patients undergoing radiotherapy with low-dose X-ray exposure exhibited elevated levels of damage markers. Aloe emodin (AE) was found to reduce radiation-induced increases in inflammatory factors IL-1β, IL-6, and TNFβ1, decrease fibrosis proportion, inhibit collagen deposition, and lower mitochondrial autophagy-related protein levels, thereby achieving cardioprotective effects. Further investigation revealed that Aloe emollient exerts multiple protective effects by reducing mitochondrial reactive oxygen species (ROS) accumulation through the PTGS2/SH3GLB1/NDP52 axis, inhibiting mitochondrial autophagy, and interfering with lactate-mediated TGF-β/Smad signaling pathway activation (Ouyang et al., 2024). The Institute of Encephalopathy at Chengdu University of Traditional Chinese Medicine proposed that Aloe emollient activates the AMPK/PGC-1α/SIRT3 pathway, regulates mitochondrial autophagy, and ameliorates early pathological changes in Alzheimer’s disease (AD) such as abnormal mitochondrial membrane potential, excessive ROS accumulation, and calcium overload, demonstrating neuroprotective effects in both *in vivo* and *in vitro* AD models (Wang Y. et al., 2025). Through clinical observation, the team led by Bian Yaoyao at Nanjing University of Chinese Medicine discovered that Huoxue Tongfu Decoction (HXTF) has potential efficacy in preventing postoperative peritoneal adhesions (PPA), with Aloe emodin being one of its primary active components. Previous validation by the authors demonstrated that FOXC2, a critical regulator of fibrotic tissue formation, is upregulated in both human ileostomy tissues and PPA rodent models, coordinating the formation of neutrophil extracellular traps (NETs) and extracellular matrix (ECM) remodeling. Using CRISPR/Cas technology, the authors found that Aloe emodin can inhibit NET formation and epithelial-mesenchymal transition by downregulating the TGFβ1-Smad2/3 signaling pathway, thereby ameliorating peritoneal fibrosis (Yang et al., 2025c).

Controllable therapeutic effects, and the lack of effective treatments for tendon-related diseases (Wang et al., 2023; Cordiano et al., 2025; Harlev et al., 2012). Regarding the downstream signaling pathways of Aloe emodin in treating peritendinous adhesion (PA), our data demonstrate that aloin can reduce the secretion of TGFβ cytokines in peritendinous tissue, inhibit the TGFβ - pSmad2/3 signaling pathway in fibroblasts, significantly suppress fibroblast activation, and thereby alleviate the progression of PA. Furthermore, considering previous literature reports, aloin exerts its effects in multiple systems such as myocardial tissue, renal tissue, and the nervous system through various mechanisms, including upregulating Smad7 expression to antagonistically inhibit Smad3 phosphorylation, reducing reactive oxygen species production to alleviate surrounding tissue inflammation, and modulating the p16 and MAPK signaling pathway. Therefore, further expanded exploration is needed to clarify the specific mechanism pathways and change trends ofAloe-emodin in tendon-related diseases (Dou et al., 2019; Zhou et al., 2021; Kam et al., 2024; Tan et al., 2025; Luo et al., 2025; Timoçin and Özen Atalay, 2026; Yin et al., 2025; Nazari et al., 2025; Humeres et al., 2024). Although Aloe emodin can alleviate the progression of PA, concerns about its potential to impair healing cannot be ignored. Through tendon biomechanical evaluation, we did not observe significant statistical differences in maximum tensile strength and Young’s modulus stiffness, indicatoing that Aloe emodin application does not interfere with the normal healing process of tendons, thus providing a guarantee fr exploring the specific potential signaling mechanisms of Aloe emodin(Supplementary Figure S9). No significant visceral toxicity was observed in the bio-compatibility test of Aloe emodin(Supplementary Figure S10).

Surgical treatment has traditionally been the first choice for peritendinous adhesion. In recent years, other biological materials, electromagnetic, and ultrasound therapies have been introduced, but their limited therapeutic modalities fail to effectively disrupt the three main and consecutive biological processes driving PA progression: physical contact between tendon and peritendinous tissue, excessive inflammation, and overactivation of fibroblasts (Titan et al., 2019; Brebels and Mignon, 2022; Ai et al., 2024; Ren et al., 2024; Xie et al., 2023; Xu et al., 2023). The limitations of many monotherapies highlight the importance of seeking a therapy that provides a physical barrier and offers efficient intervention throughout the entire course. Ultrasound and electromagnetic therapies are non-invasive and convenient, but cannot provide a physical barrier. Some drugs, such as mitomycin C(MMC), can early inhibit fibroblast proliferation or even induce apoptosis, but excessive local injection instead exacerbates scarring and may lead to non-healing wounds, making their efficacy uncertain and unsuitable as ideal drugs (Sultana et al., 2020; Titan et al., 2019; Brebels and Mignon, 2022; Ai et al., 2024; Ren et al., 2024; Xie et al., 2023; Xu et al., 2023; Song et al., 2024; Xie et al., 2025; Dai et al., 2025). Traditional electrospun membranes, due to their porous characteristics, have been widely used for drug co-delivery. However, the burst release of drugs and the uncertainty of release location make it difficult to achieve precise therapeutic application and long-term efficacy (Kam et al., 2024; Tan et al., 2025). In this study, we combined aloin with polylactic acid materials through esterification reaction using conjugation technology. Both nuclear magnetic resonance and Fourier transform infrared spectroscopy confirmed the success of the esterification reaction. Compared with traditional techniques, after conjugation, the composite material electrospun into a membrane can be continuously hydrolyzed by lipase secreted by damaged tissues at the injured site in rats, enabling prolonged drug release. Molecular biological studies demonstrated that aloe emollient can inhibit the TGFβ classical activation pathway through the TGFβ1-pSmad2/3-COLIII pathway in post-tendon injury adhesions, thereby exerting anti-fibrotic and anti-peritendinous adhesion effects. Protein level changes in rat peritendinous tissues indicated that compared to simple drug mixtures, polylactic acid-Aloe emodin graft materials could achieve targeted controlled release at the lesion site, significantly prolonging the therapeutic time window. In addition to fibroblasts, Aloe emodint treatment markedly reduced the secretion levels of inflammatory cytokines (TNFα, IL-1β, IL-6) by macrophages. This precisely intervenes in the local environment of the damaged tendon and reduces PA progression. Drug release tests confirmed that under the action of lipase, the PCA membrane had a high release amount of aloin in the first 10 days, and still exhibited sustained and efficient drug release performance in later release tests. It showed anti-adhesion effects covering the early, middle, and late stages in rat tendon adhesion.

This study successfully prepared polylactic acid-Aloe emodin conjugates by grafting aloe emodin onto polylactic acid through esterification. A novel polylactic acid-Aloe emodin conjugated electrospun nanofiber membrane was fabricated and characterized using electrospinning technology. Systematic validation demonstrated its potential to prevent peritendinous adhesions. However, our experiments in this study also have many limitations. First, the newly generated nanofiber membranes themselves are laboratory-synthesized materials. In actual large-scale production applications, their specific quality and performance will be constrained by factors such as the source of chemical raw materials, the proficiency of the synthesis process, and the control of reaction conditions, making it difficult to achieve a stable and ideal product supply. Second, the sample size and observation time points for *in vivo* and *in vitro* experiments can still be optimized. More observation windows can help us relatively comprehensively evaluate the stability of PCA materials in long-term implantation and complex *in vivo* environments. Finally, due to the limited availability of rat tendon sheath materials, our exploration of the specific molecular mechanisms by which Aloe emodin downregulates TGFβ signaling, inhibits type III collagen secretion, and improves tendon sheath adhesion prognosis is insufficient (Humeres et al., 2024; Miyazawa and Miyazono, 2017; Wang et al., 2014). Compared to existing literature reports, Aloen emodin can influence the occurrence and development of fibrotic diseases from multiple angles, such as anti-inflammatory, antioxidant, and anti-fibrotic effects. Our current study has not achieved a comprehensive analysis of the multi-pathway mechanism of Aloe emodin in promoting tendon healing (Yong et al., 2024; Chen et al., 2025). The emergence of these issues suggests that further exploration is needed for the future application of this new grafted material.

## 4 Conclusion

This study achieved the generation of polylactic acid-Aloe emodin conjugate compounds by grafting Aloe emodin onto polylactic acid through esterification reactions. Using electrospinning technology, novel polylactic acid - Aloe emodin conjugate electrospun nanofiber membranes were prepared and characterized. Systematic validation demonstrated their role in preventing peritendinous adhesions. Experiments showed that the polylactic acid-Aloe emodin grafted material can significantly alter the original morphological characteristics of pure polylactic acid electrospun membranes and enhance the mechanical properties of nanofibers, serving as an effective carrier for postoperative peri-tendinous adhesion intervention. PCA exhibited good anti-adhesive effects early on and had more sustained efficacy compared to PBA, achieving the goal of controlled drug release and extended treatment windows for electrospun nanofiber membranes. *In vitro* experiments indicated that PCA effectively downregulates fibroblast activity, inhibits their proliferation, adhesion, and PA formation. *In vivo* experiments verified that PCA could wrap around surgical tendons and inhibit the TGF β1 - pSmad2/3 - Col III signaling pathway, proposing a new direction for PA treatment.

## 5 Experimental section

Materials: Polylactic acid (PLA, Mw = 80,000) was obtained from Rhawn Chemicals Co., Ltd. (Shanghai, China). Aloe emodin was obtained from Shanghai Yuanye Biotechnology Co., Ltd. 1-(3-dimethylaminopropyl)-3-ethylcarbodiimide hydrochloride (EDCI), and 4-dimethylaminopyridine (DMAP) were purchased from Shanghai Aladdin Biochemical Technology Co., Ltd. 1,1,1,3,3,3-hexafluoro-2-propanol (HFIP) was obtained from Da-Rui FineChemical Co., Ltd. Fetal bovine serum (FBS) and penicillin-streptomycin double-antibody solution were purchased from Thermo Fisher Scientific, USA. Calcein/PI cell viability and cytotoxicity detection kits, Edu staining kits, CCK-8 detection kits, hematoxylin-eosin (HE) staining kits, Masson tricolor staining kits, BCA kits, and TNF-α/IL-1β/IL-6 ELISA detection kits were all purchased from Shanghai Biocytogen Biotechnology Co., Ltd. in China. Phalloidin staining kits were purchased from MCE, USA. TGFβ1/COLIII/GAPDH primers were all purchased from Shanghai Guantai Biotechnology Co., Ltd. in China. qPCR reverse transcription kits were purchased from Takara, Japan. TGFβ1, Smad2/3, and pSmad2/3 rabbit anti-rat primary antibodies were purchased from Abogen, China. DMEM high-sugar medium, COLIII rabbit anti-rat primary antibody, β-Aactin goat anti-rabbit direct-label internal standard antibody, ECL chemiluminescence kit, and mIHC immunohistochemical double-staining kit were purchased from Wuhan Savill Biotech Co., Ltd. in China.

PLA-Aloe emodin conjugate material synthesis: We synthesized the PLA-aloe-emodin conjugate through esterification reaction. In a round-bottom flask equipped with a magnetic stirrer, 3 g of PLA and 0.3 g of aloe-emodin were dissolved in 50 mL of dichloromethane. The entire process must be carried out under nitrogen protection. Subsequently, 0.204 g of EDCI and 0.086 g of DMAP were added to the flask, and it was stirred continuously at room temperature for 12 h. The reaction mixture was then poured into a mixed solution of ethanol and deionized water to precipitate the product, which was repeatedly dissolved and precipitated three times with dichloromethane. Finally, vacuum drying was performed to obtain the grafted material.

The preparation and characterization of nanomaterials: Nanomaterials were synthesized via electrospinning. The brief process involves dissolving PLA-aloe emodin conjugates at a concentration of 15 w/v% in HFIP at room temperature. The injection speed of a blunt needle (20G) was set to 1.5 mL h−1, and the voltage was set to 13–15 kV. Nanofibers were collected using a slowly rotating mandrel wrapped with aluminum foil (diameter 6 cm, speed 120 rpm) at a distance of 10 cm from the needle tip. To obtain PLA nanofiber membranes doped with aloe emodin, the dissolution ratio of PLA in HFIP was set to 15%, and the dissolution ratio of aloe emodin in the PLA solution was set to 1.5%, followed by electrospinning under the same conditions as above. Electrospinning was also performed using pure PLA solution to prepare PLA nanofiber membranes. Pure PLA, PLA doped with aloe emodin, and PLA electrospun membranes grafted with aloe emodin are referred to as PLA, PBA, and PCA, respectively. The morphology of the nanomaterials was observed by scanning electron microscopy using the Phenom XL from Phenom-World at an acceleration voltage of 5 kV after gold coating, before vacuum drying(SC7620, Quorum, England). The chemical structure was analyzed by attenuated total reflection mode of Nicolet-is10 FTIR spectrometer in the wavelength range of 4,000 to 400 cm−1. The surface wettability of the nanomaterials was determined by measuring the water contact angle using the Dataphysics DCAT21 contact angle analyzer from Germany. After drying the nanomaterials in a vacuum oven, 5 μL of deionized water was gently dropped onto each membrane surface, and the tangent of the water droplet surface on different nanomaterials was constructed for calculation (n = 6). The tensile properties of the nanomaterials were determined by uniaxial tensile testing as previously described. The nanomaterials were cut into 10 × 50 mm strip samples for testing. The samples were mounted in the grips of a uniaxial testing machine (Instron 5,966, INSTRON, American) equipped with a 50 N load cell and then stretched at a speed of 10 mm per minute until the nanomaterials broke. All data were obtained from six replicates and used to calculate the ultimate tensile strength (UTS) before maximum load, and the Young’s modulus was calculated from the slope of the initial 5% linear portion of the stress-strain curve (n = 6).

Cell culture: Obtain the 208F rat fibroblast cell line from ATCC and culture it in standard complete DMEM medium. This medium consists of 94% DMEM, 5% fetal bovine serum, and 1% penicillin-streptomycin. ENMs are cut into circular pieces similar in diameter to those of a 24-well plate and sterilized under UV light for 2 h in a laminar flow hood before seeding. The 208F cells are seeded at a density of 1 × 10^4^ cells per well in pre-treated 24-well plates for live/dead staining, cytoskeleton scanning, and EDU proliferation detection, and at a density of 3 × 10^3^ cells per well in 96-well plates for CCK8 assay.

*In Vitro* Drug Release Determination: The PBD and PCD were cut into strip shapes weighing 20 mg, and then soaked in 3 mL 1*PBS under constant 37 °C. They were divided into two groups, one treated with 0.05 mg/mL lipase and the other untreated. Every 2 days, 3 mL of solution was collected and replenished with the same volume of PBS. The samples were adjusted to 0.5 mol/L with NaOH and then measured. The release of aloe-emodin was determined using a UV-Vis spectrophotometer (UV-2700, Shimadzu) at a wavelength of 276 ± 2 nm. A standard curve was plotted using standard samples of 0, 5, 10, 15, 20, and 25 μg/mL and their absorbance values (R2 = 0.9978) (Supplementary Figure S6). For CCK8 assay, 208Fs were cultured on 24-well plates pre-treated with different fiber membranes for 1, 4, and 7 days, with or without lipase treatment in each group. At the predetermined observation points, cells on the 24-well plates were digested, transferred to 96-well plates, allowed to adhere, and then 10 μL of CCK8 solution was added to each well for co-culture for 1 h. Absorbance was then measured using a spectrophotometer at 450 nm.

Cell proliferation and adhesion assessment: On day 1 and day 4, fibroblasts in 24-well plates pre-treated with PLA, PCD, and PBD (all containing lipase) were stained with live/dead stain to estimate the viability of 208Fs. The live/dead staining was performed using a Live/Dead kit, and the results were analyzed using ImageJ and assessed by the ratio of dead to live cells. Phalloidin staining was carried out using a Phalloidin kit to observe the arrangement of the cytoskeleton. Brief steps include: treating the 208Fs in PLA, PCD, and PBD groups with 4% paraformaldehyde for 30 min, followed by treatment with 0.2% Triton X-100 for 10 min. Prepare a phalloidin solution at 20 μg mL−1, add 200 μL to each well, then add 1 μg mL−1 DAPI solution. Images were collected using a fluorescence microscope (BZ-X800E, KEYENCE) and statistically analyzed using ImageJ. Fibroblasts were seeded in 24-well plates, typically using logarithmic growth phase cells, suspended to 2 × 10^5^ cells/mL, and 200 μL of cell suspension was added to each well, incubated in a 37 °C incubator. According to experimental needs, corresponding treatments and stimuli were performed to allow normal growth to the desired density. After removing 100 μL of the original cell culture medium, the EdU working solution was diluted to 20 μM, 100 μL of the EdU working solution was added to each well, diluted to a concentration of 10 μM, and incubated at 37 °C for 3 h. The culture medium was discarded, and the cells were washed once or twice with PBS. 200 μL of 4% paraformaldehyde was added to each well to fix the cells for 30 min. Cells were permeabilized with PBS containing 0.5% Triton X-100, typically for 20 min 200 μL of the EdU reaction mixture was added to each well, incubated in the dark at room temperature for 30 min to allow the fluorescent dye to bind to EdU. The cells were washed once or twice with PBS, and the nuclear stain was diluted to 1:1,000 in PBS, 200 μL was added to each well for nuclear staining. Incubate in the dark for 10 min. Finally, quantitative analysis of cell proliferation was performed using a fluorescence microscope (BZ-X800E, KEYENCE) and ImageJ.Rat Achilles Adhesion Model: All the rat experiments accorded with the guidelines published by NIH and were approved by Shanghai Jiao Tong University (approval no. SYXK(Hu)2016–0,020). A total number of 96 male rats (Sprague–Dawley) weighing 250–300 g were randomly divided into two groups according to the time points of 3 and 6 weeks, and each group was split into four groups (n = 12 per group): the control group, PLA group, PCD group, and PBD group. Specifically, the rats were inhalational anesthetized with isoflurane and then disinfect the hind limbs with 75% ethanol. Make an incision of skin to expose the Achilles tendon, and then transect the tendon and repair it by Kessler’s suture method. The PLA, PCD, and PBD were cut into 0.5 × 1 cm pieces to wrap around the sutured tendon, and the control group was treated with no ENM.

Rat Achilles Adhesion Model: All rat experiments complied with guidelines released by the National Institutes of Health (NIH) and were approved by Shanghai Jiao Tong University (approval number: A2025392). A total of 48 male Sprague–Dawley rats, each weighing approximately 250–300 g, were used. They were randomly divided into four groups based on two time points, 3 weeks and 6 weeks, and each group was further divided into four subgroups (six rats per subgroup): control group, PLA group, PBA group, and PCA group. The specific procedure involved administering inhalation anesthesia to the rats using isoflurane, disinfecting the hind limbs with 75% ethanol. The skin was incised to expose the Achilles tendon, which was then cut and repaired using the Kessler suture method. PLA, PBA, and PCA were cut into 0.5*1 cm pieces and wrapped around the sutured Achilles tendon, while the control group did not use nanofiber membranes.

Gross Evaluation:Signs of inflammation or infectious ulcers of rat limbs were observed at 3 weeks and 6 weeks post-surgery. Make a longitudinal incision in the skin to expose the surgical site. Grade the results based on observations and assessments.

Histological assessment: After perfusion with 4% paraformaldehyde, the treated Achilles tendon was surgically removed and further fixed with 4% paraformaldehyde for 24 h. The Achilles tendon tissue block was then immersed in a horizontal bidirectional oscillator containing a 25% ethylenediaminetetraacetic acid (EDTA) solution for 2 months for decalcification. The decalcified limb was embedded through a standard paraffin embedding process, and all paraffin specimens were sectioned into 5-μm-thick slices according to the sagittal position. Histological assessment was performed using hematoxylin-eosin staining, Masson’s trichrome staining, and immunohistochemical staining. Images were scanned using a Leica microscope (DMI6-M, Leica Microsystems). The degree of adhesion at the surgical site was estimated on a scale of 1–5 (Figure S, Supplementary Material). The degree of healing at the surgical site was estimated on a scale of 1–4 (Supplementary Figure S11, Supplementary Material). The primary antibodies used in this study were as follows: anti-Col III (Arigo, ARG20786, 1:2000).

The macroscopic evaluation standards for peritendinous adhesion. The macroscopic evaluation standards was evaluated into Grade 1–5 based on the surgical observation: Grade 1, no obvious adhesions around tendon; Grade 2, a little adhesion tissue could be easily separated from the Achilles tendon; Grade 3, less than 50% of the adhesion area is separable using a blunt instrument from the Achilles tendon; Grade 4, 51%–97.5% of the adhesion area is separable using a blunt instrument from the Achilles tendon; Grade 5, more than 97.5% of the adhesion area is separable using a blunt instrument from the Achilles tendon.

The histological evaluation standards for peritendinous adhesion. The histological scoring of adhesion tissue was evaluated into Grades 1–5. Grade 1, no adhesions; grade 2, adhesion tissue holding less than 25% in the peritendinous area of the repaired site; grade 3, adhesion tissue holding less than 25%–50% in the peritendinous area of the repaired site; grade 4, adhesion tissue holding 50%–75% in the peritendinous area of the repaired site; grade 5, adhesion tissue holding more than 75% in the peritendinous area of the repaired site.

The histological evaluation standards for tendon healing. The histological scoring for tendon healing was quantified into Grades 1–4. Grade 1, good tendon continuity and smooth epitenon surface; grade 2, intratendinous collagen bundles exhibited good repair, but the epitenon was interrupted by adhesions; grade 3, irregularly arranged and partly broken intratendinous collagen bundles. Grade 4, failed healing or massive overgrowth of granulation tissue.

Biomechanical testing: A rheometer (Instron 5,569; Instron, Norwood, Massachusetts, USA) to test the maximum tendon breaking strength, thereby quantifying tendon healing assessment. The proximal end is marked as the calcaneus, and the distal end is marked as the tendon-muscle part of the hind claw, using two metal force gauges to stretch them in opposite directions at a speed of 30 mm per minute until rupture (Figure S).

Poly chain reaction (PCR):Total RNA was extracted from the tendon surrounding tissue and cell lines using an RNA extraction kit. The purity and concentration of RNA were measured using a NanoDrop spectrophotometer (Thermo Fisher Scientific Inc.). Reverse transcription was performed using PrimeScript™ RT Master Mix to obtain complementary DNA (cDNA). A 20 µL real-time PCR mixture included SYBR Green PCR Master Mix, upstream and downstream primers, cDNA samples, and nuclease-free water. The specific real-time PCR amplification conditions were as follows: initial denaturation at 94 °C for 30 s, followed by 40 cycles, each cycle including denaturation at 94 °C for 10 s, and annealing at 60 °C for 30 s. The accumulation of PCR products was detected using the ABI PRISM® 7500 Sequence Detection System (Applied Biosystems, California, USA). The cycle threshold (Ct value) was reported when the SYBR green fluorescence exceeded the threshold. Relative mRNA expression was calculated using the 2^−ΔΔCT^ method and normalized to the housekeeping gene GAPDH.

ELISA: The cultured macrophages were resuspended by pipetting to prepare a cell suspension. After cell counting, 1 × 10^5^ cells were inoculated into 24-well plates, divided into control, PLA-treated group, PBA-treated group, and PCA-treated group. Each group consisted of 6 replicates, with 500 μL of complete medium (containing LPS added proportionally to a final concentration of 100 ng/mL) added per well. The plates were incubated in a 37 °C, 5% CO2 incubator. Co-culture of macrophages with electrospun fibrous membranes was maintained for 4 days before experimental termination. The cell culture supernatant was collected and centrifuged (1,000 rpm, 5 min). The ELISA Kit reagents were used to prepare the standard solution according to the protocol, followed by biotinylated antibody incubation, horseradish peroxidase labeling, and TMB chromogenesis to detect the levels of relevant inflammatory factors in the macrophage supernatant.

Weston Blot(WB): After co-incubation of Aloe emodin and LPS, directly add protein lysate (RIPA Strong Lysate, stock number G2002-100 mL) to the culture dish, followed by the addition of protease inhibitor (G2008-1 mL) and phosphatase inhibitor (G2007-1 mL) in the specified proportions. Use a cell scraper to collect the cells, then place the dish on ice or in a 4 °C refrigerator for 20–30 min to lyse the cells. Collect the lysate in a centrifuge tube and centrifuge (12,000 rpm, 4 °C pre-cooled, 20 min). Pipette the supernatant into a new centrifuge tube to obtain the protein extract. For animal tissue samples, thaw the frozen tissue from the-80 °C refrigerator, cut a sample of approximately 20 mg per portion, and place it in a centrifuge tube. Add RIPA Strong Lysate at a ratio of 500 μL/20 mg, followed by the addition of phosphatase and protease inhibitors as specified in the protocol. Add two magnetic beads to each centrifuge tube and mix thoroughly. Grind the tissue in a low-temperature tissue grinder. After grinding, incubate the tissue on ice for 20 min to ensure complete lysis, then centrifuge for 20 min. Transfer the supernatant to a new centrifuge tube to obtain the animal tissue protein extract. Use protein quantification (BCA method) to determine the protein concentration of the sample. After boiling, gel preparation, loading, electrophoresis, membrane transfer, blocking, primary antibody incubation, secondary antibody incubation, and development, perform grayscale analysis of protein bands using ImageJ.

Statistical Analysis: All experiments were conducted as 6 repetitions with the mean ± standard deviation (SD) representing for value. All the charts and data were analyzed by GraphPad Prism10 and ImageJ adhered to principles of statistics as the Student’s t-test for comparison of two groups and one-way analysis of variance (ANOVA) with Fisher’s exact tests for multiple comparisons. Prior to parameter testing, we conducted a Shapiro-Wilk test using Prism software to assess the normality distribution of the data. In this study, a p-value <0.05 was considered significantly different.

## Data Availability

The original contributions presented in the study are included in the article/Supplementary Material, further inquiries can be directed to the corresponding authors.
