# Supplementary figures and images for: Therapeutic effect of sustained release of aloe emodin via novel grafted polymer in electrospun nanofiber membranes on peritendinous adhesions
Source: Front Bioeng Biotechnol. 2026 Apr 21;14:1780630. doi: 10.3389/fbioe.2026.1780630 (PMC13139084; doi:10.3389/fbioe.2026.1780630)

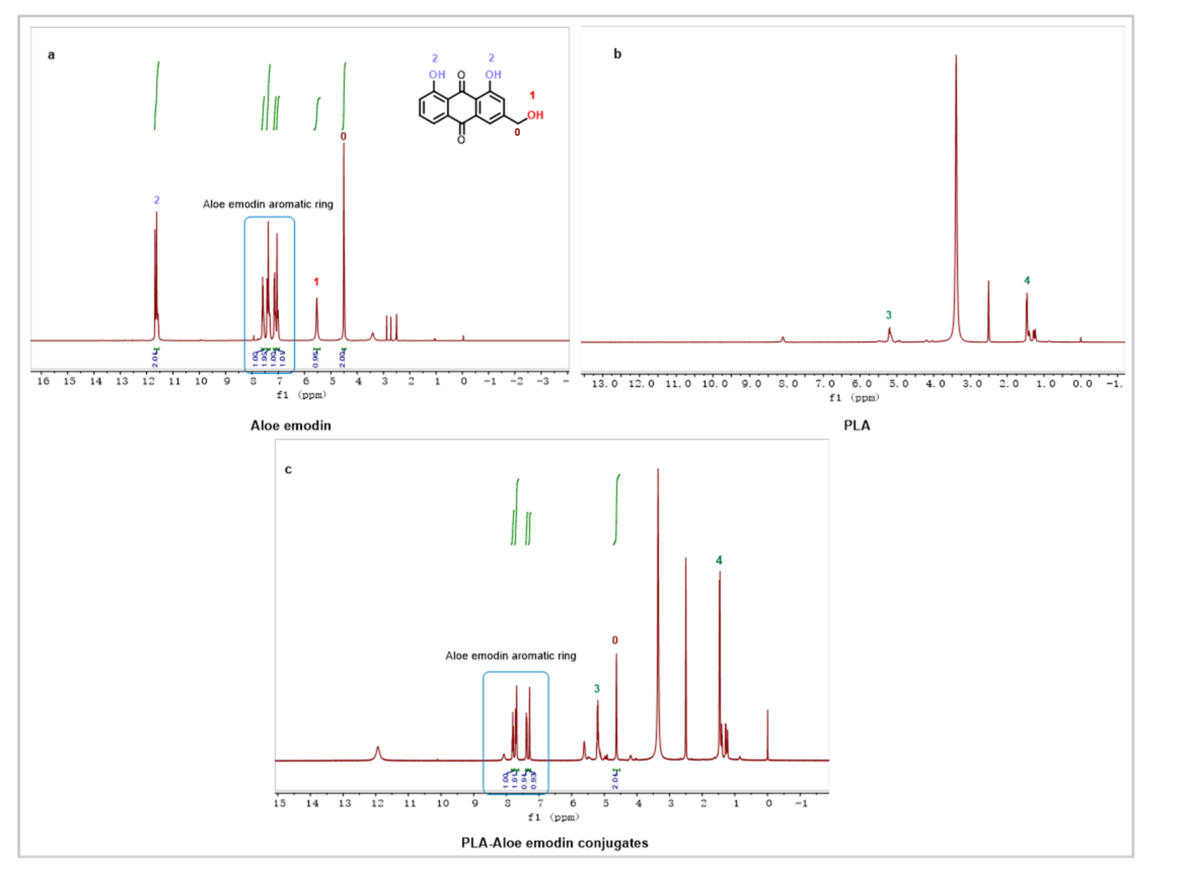

Supplement: Supplementary file 1 [file Image3.jpeg]

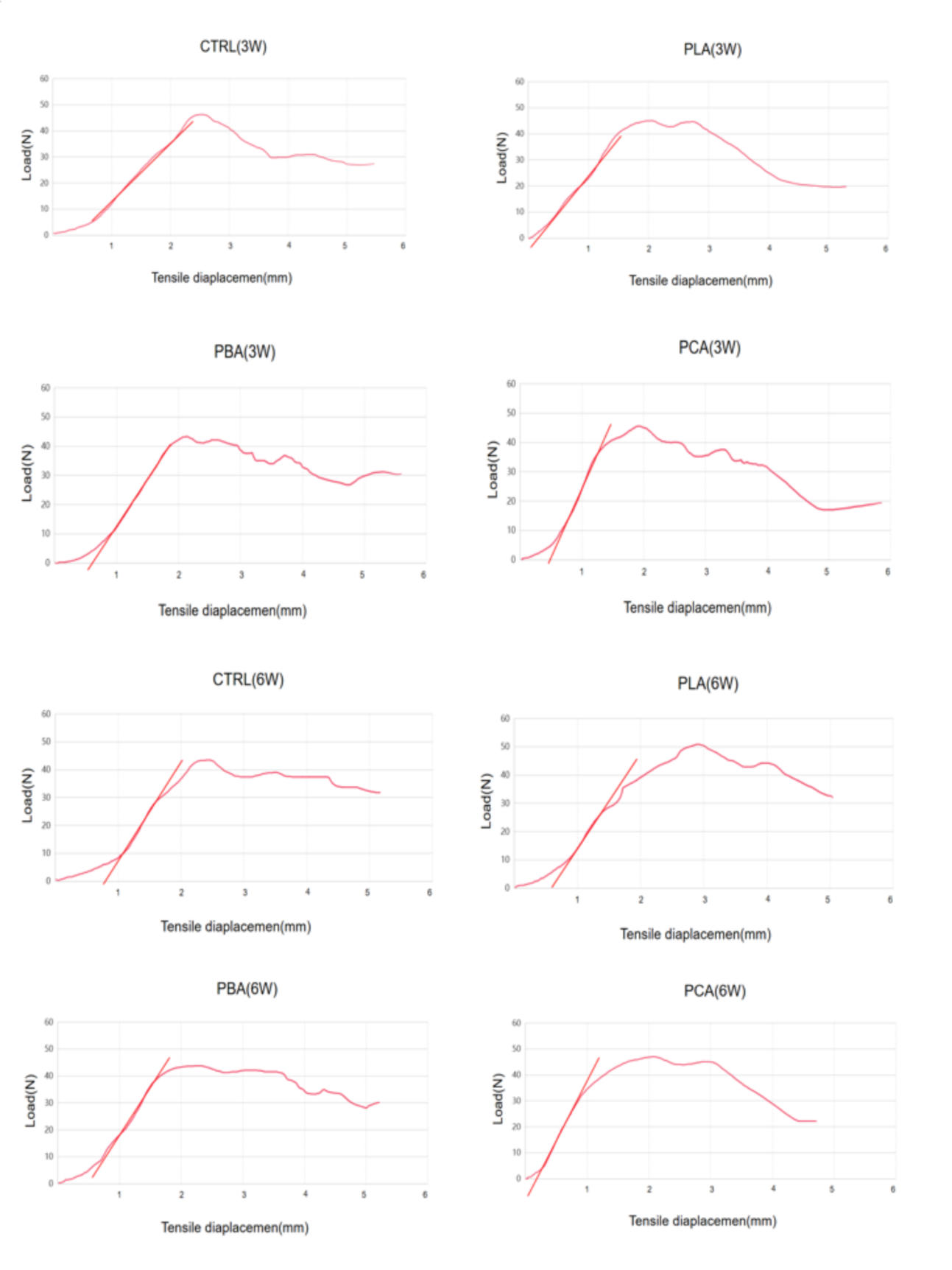

Supplement: Supplementary file 2 [file Image9.jpeg]

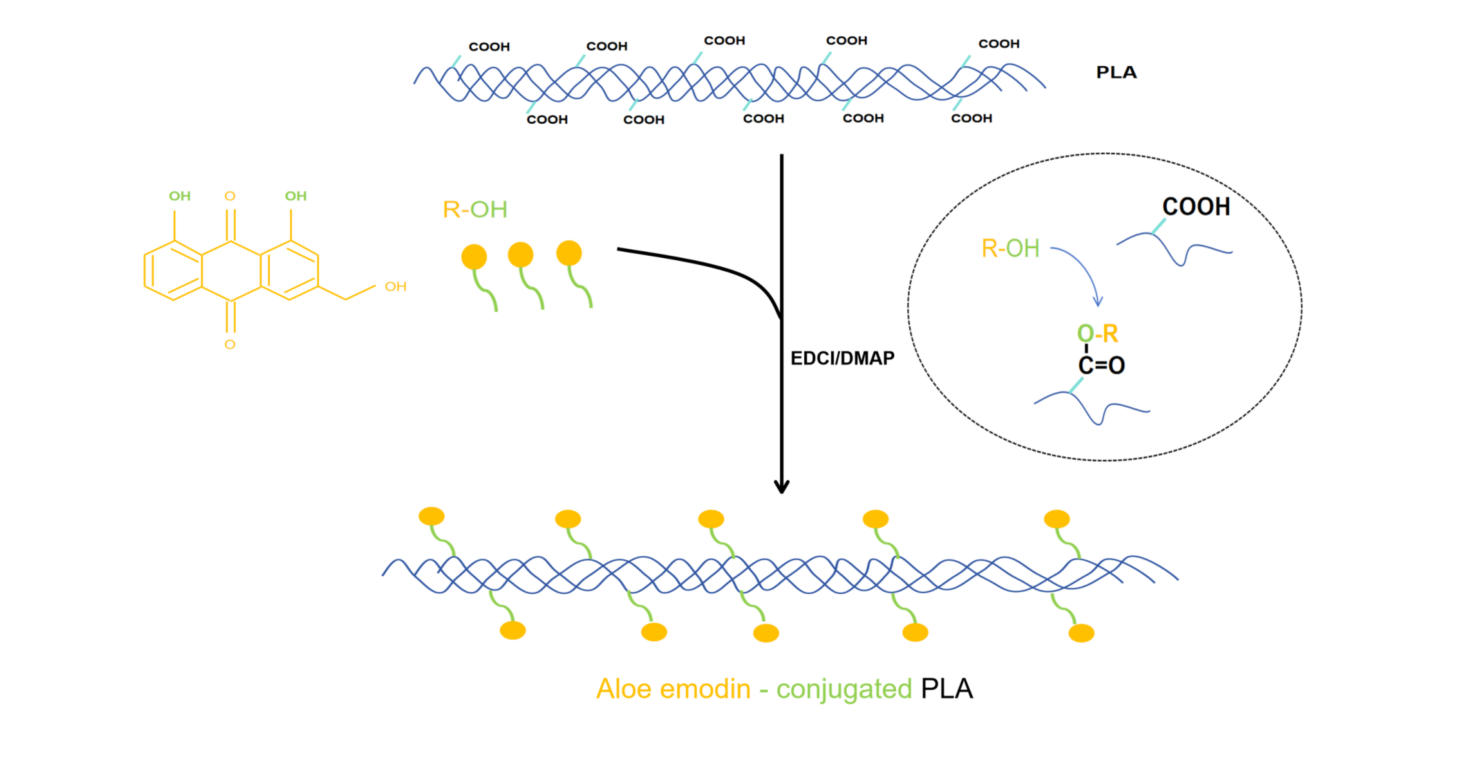

Supplement: Supplementary file 3 [file Image1.jpeg]

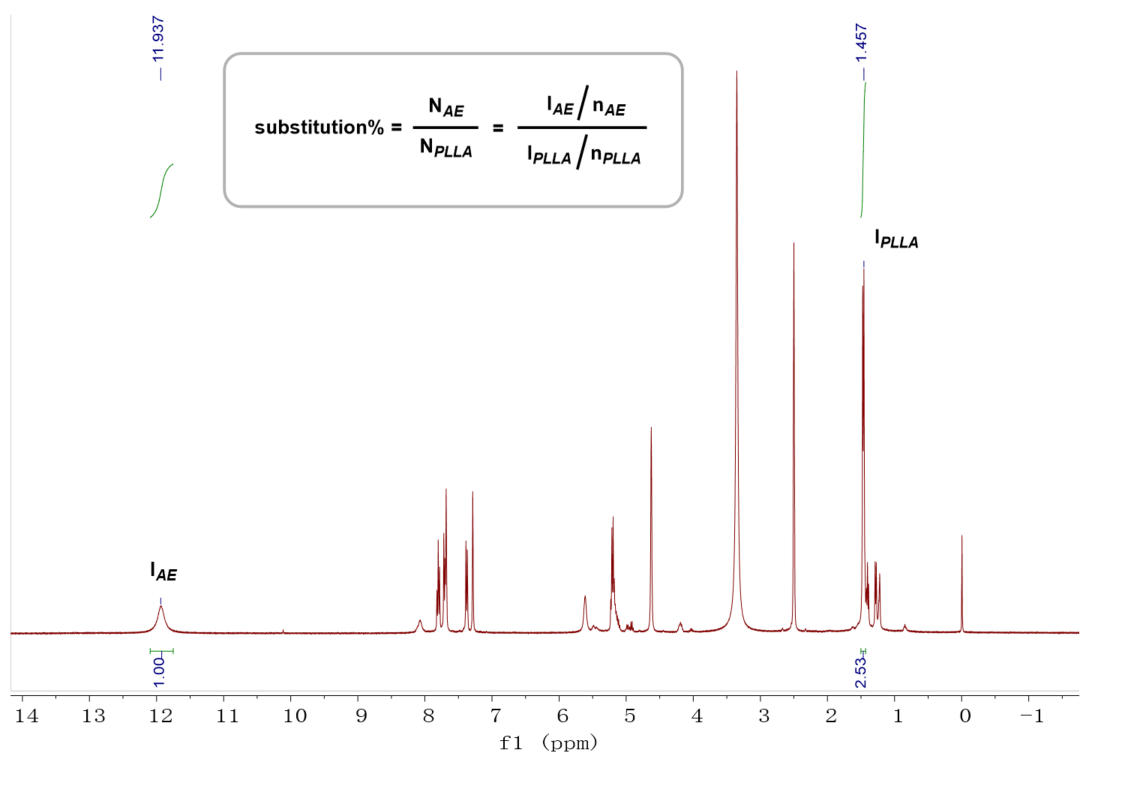

Supplement: Supplementary file 4 [file Image4.jpeg]

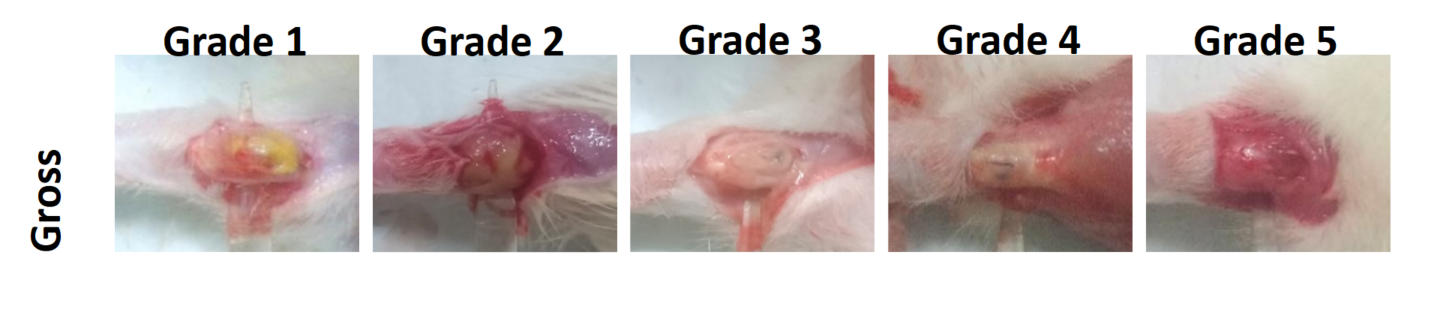

Supplement: Supplementary file 5 [file Image7.jpeg]

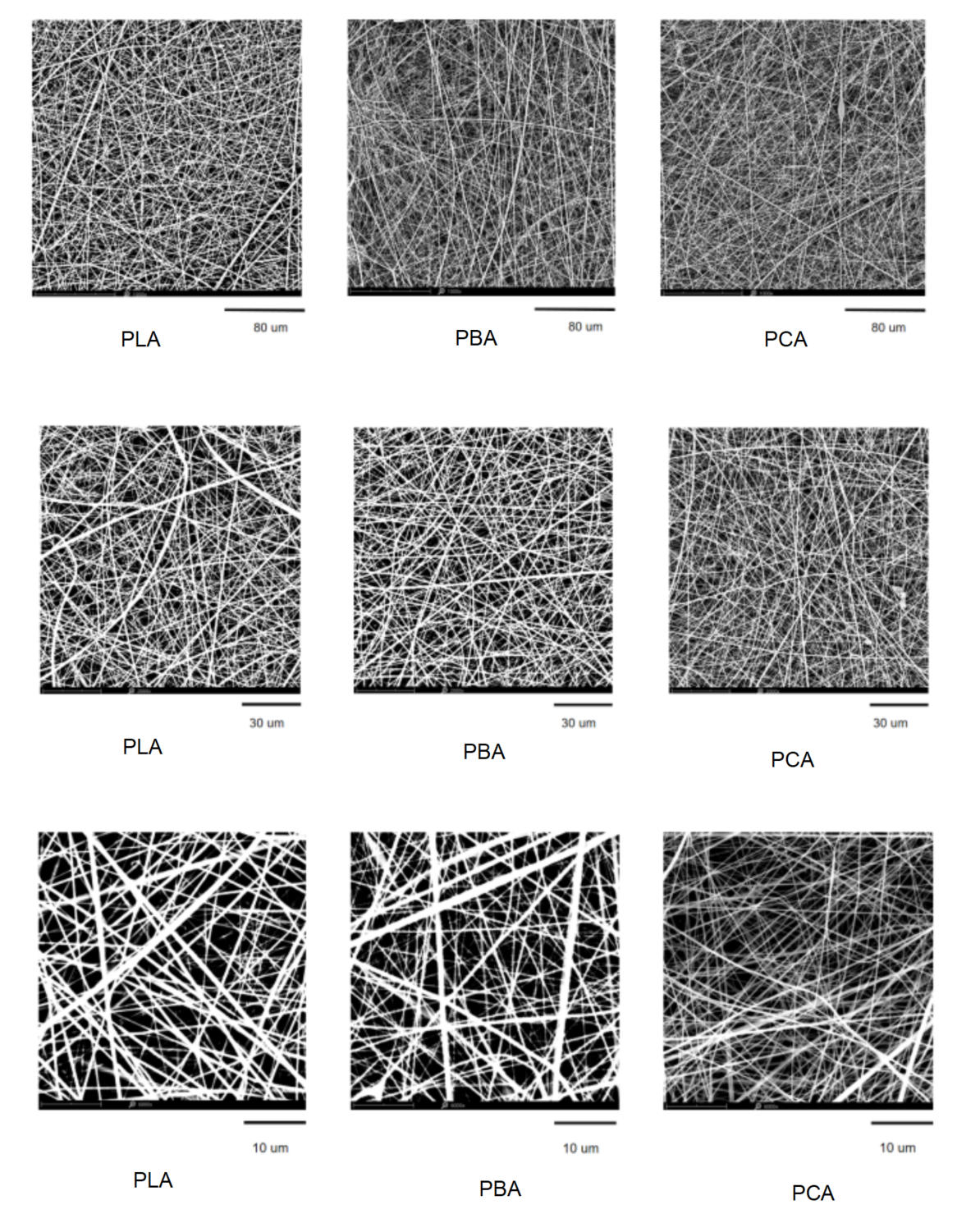

Supplement: Supplementary file 6 [file Image2.jpeg]

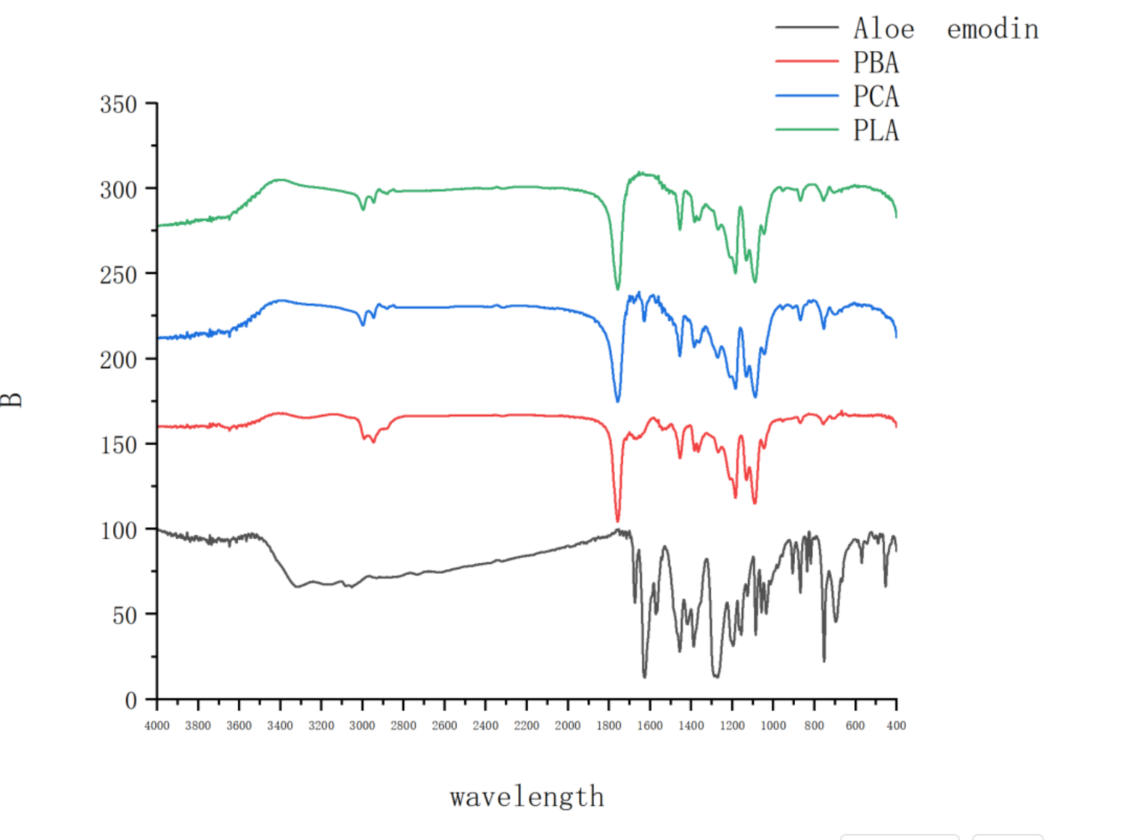

Supplement: Supplementary file 7 [file Image5.jpeg]

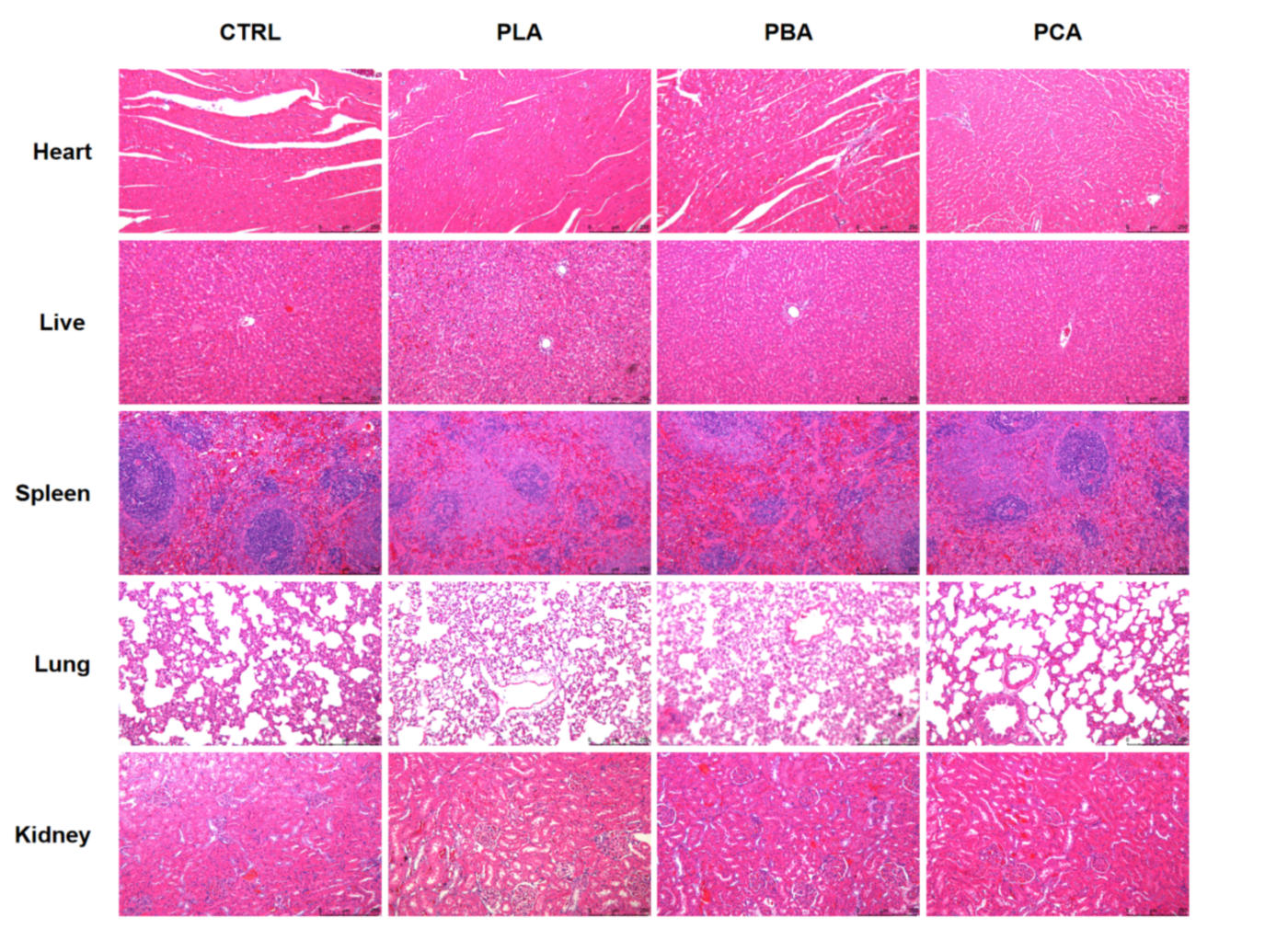

Supplement: Supplementary file 8 [file Image10.jpeg]

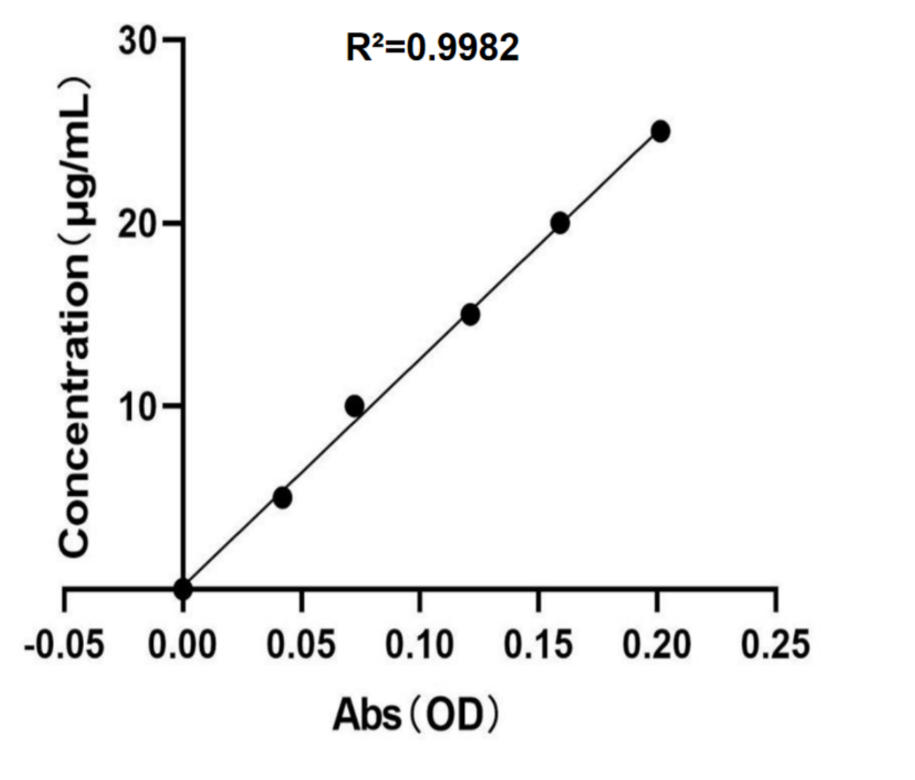

Supplement: Supplementary file 9 [file Image11.jpeg]

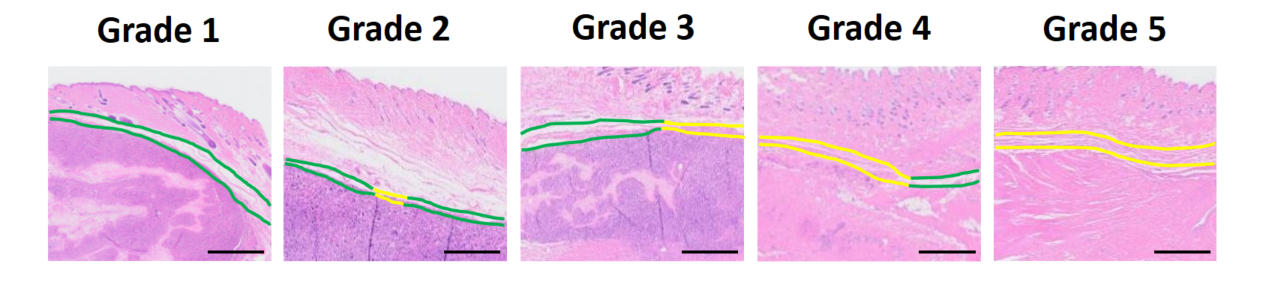

Supplement: Supplementary file 10 [file Image8.jpeg]

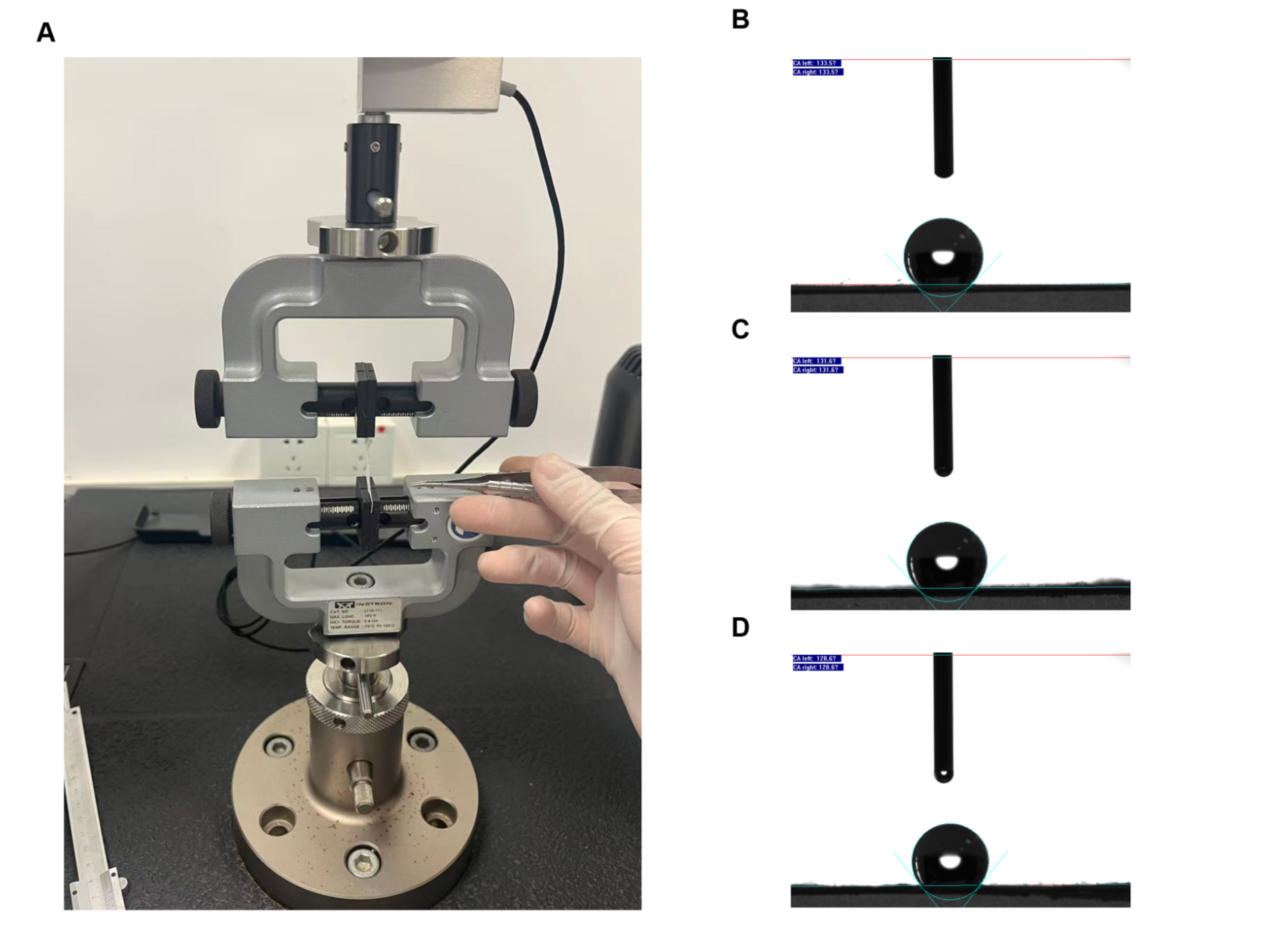

Supplement: Supplementary file 11 [file Image6.jpeg]
